# Supplementary material for: Infection with Mycobacterium tuberculosis alters the antibody response to HIV-1
Source: PLoS Pathog. 2025 Aug 13;21(8):e1013350. doi: 10.1371/journal.ppat.1013350 (PMC12370194; doi:10.1371/journal.ppat.1013350)
Supplement: S1 Table — (originally published by Rusert et al. 2016, Nature Medicine, doi: https://doi.org/10.1038/nm.4187) [45]. (DOCX) [file ppat.1013350.s001.docx]

## **S1 Table HIV-1 virus panel for neutralization assay** (originally published by Rusert et al. 2016 Nature Medicine, (1), doi: [10.1038/nm.4187)](https://doi.org/10.1038/nm.4187)

| **HIV-1 subtype** | **Virus** | **GenBank entry code** |
| --- | --- | --- |
| A | BG505_W6M_ENV_A5_T332N | DQ208456 |
| A | KER2018.11 | AY736810 |
| A | Q23_17 | AF004885 |
| AE | CNE59 | HM215422 |
| B | JR-FL | AY669728 |
| C | HIV_25925_2 | EF117273 |
| C | Du156.12 | DQ411852 |
| G | NAB13pre_cl_9 | EU023937 |

**Reference**

1. Rusert P, Kouyos RD, Kadelka C, Ebner H, Schanz M, Huber M, et al. Determinants of HIV-1 broadly neutralizing antibody induction. Nat Med [Internet]. 2016;22(11):1260–7. Available from: https://doi.org/10.1038/nm.4187
