# Supplementary material for: Infection with Mycobacterium tuberculosis alters the antibody response to HIV-1
Source: PLoS Pathog. 2025 Aug 13;21(8):e1013350. doi: 10.1371/journal.ppat.1013350 (PMC12370194; doi:10.1371/journal.ppat.1013350)
Supplement: S2 Table — (originally published by Liechti et al. 2018, Journal of Immunological Methods, doi: https://doi.org/10.1016/j.jim.2017.12.003) [43]. (DOCX) [file ppat.1013350.s002.docx]

## **S2 Table HIV-1 antigens for binding assay** (originally published by Liechti et al. 2018, Journal of Immunological Methods, (1), doi: <https://doi.org/10.1016/j.jim.2017.12.003>)

| **Antigen** | **HIV strain** | **HIV-1 subtype** | **Additional information**  **(source, reference, gene association number, sequence)** |
| --- | --- | --- | --- |
| p17 | NL4-3 | B | GenBank: AF324493., p17 CDS: 790..18 |
| p24 | BH10 | B | Purchased from Aalto Bio reagents (CatNo AG6054) |
| JR-FL gp120 | JR-FL | B | GenBank: AAB05604.1, gp120 CDS: 73..1491 (aa: 25-497) |
| BG505 trimer (SOSIP.664) | BG505 | A | The construct is based on the BG505 (BG505.W6M.ENV.C2) env gene (GenBank accession nos. ABA61516 and DQ208458, DOI: doi:10.1128/JVI.80.2.835–844.2006) and was originally obtained from John P. Moore (Cornell University) and Rogier Sanders (Amsterdam Medical Center). DOI: 10.1371/journal.ppat.1003618; construct contains T332N |
| BG505 gp140 | BG505 | A | Same construct as above. Monomer is obtained through separation by size exclusion chromatography. |
| RSC3 | HXB2 | B | Original expression plasmid provided by Peter D. Kwong (Vaccine  Research Center, NIH); DOI: 10.1126/science.1187659 |
| RSCΔ |  |  |  |
| V3 JR-FL | JR-FL | B | Provided by John Robinson (University of Zurich) linear peptide: "NNTRKSIHIGPGRAFYTTGEIIG" |
| V3 MN | MN | B | Provided by John Robinson (University of Zurich)  linear peptide: "GGGGYNKRKRIHIGPGRAFYTTKNIIG" |
| V3 BG505 | BG505 | A | Provided by John Robinson (University of Zurich), linear peptide: "EKSIRIGPGQAFYAT" |
| MPER-2/4 | MN | B | MPER peptide encompassing the epitopes of MPER bnAbs 2F5 and 4E10. Provided by John Robinson (University of Zurich) sequence: "EQELLELDKWASLWNWFDITNWLWYIR" |
| MPER-2/4/10 | MN | B | MPER peptide encompassing the epitopes of MPER bnAbs 2F5,  4E10 and 10E8. Provided by John Robinson (University of Zurich)  sequence: "EKNEQELLELDKWASLWNWFDITNWLWYIK" |
| gp41ΔMPER | SF162P3 | B | Recombinant trimerized gp41 ectodomain from strain SF162P3 (aa540-665 HXB2 numbering; GenBank: AY988107). Expression plasmid provided by James Voss and Dennis Burton (The Scripps Research Institute, La Jolla, USA). |

**Reference**

1. Liechti T, Kadelka C, Ebner H, Friedrich N, Kouyos RD, Günthard HF, et al. Development of a high-throughput bead based assay system to measure HIV-1 specific immune signatures in clinical samples. J Immunol Methods. 2018 Mar;454:48–58.
