# Supplementary material for: Infection with Mycobacterium tuberculosis alters the antibody response to HIV-1
Source: PLoS Pathog. 2025 Aug 13;21(8):e1013350. doi: 10.1371/journal.ppat.1013350 (PMC12370194; doi:10.1371/journal.ppat.1013350)
Supplement: S3 Table — Abbreviations: MTB (Mycobacterium tuberculosis), CMV (Cytomegalovirus), Toxoplasma (Toxoplasma gondii), and HCV (Hepatitis C virus). (DOCX) [file ppat.1013350.s003.docx]

**S3 Table Demographic characteristics by serostatus to various diseases**

|  | **MTB uninfected** | **MTB asymptomatic** | **IGRA MTB uninfected** | **IGRA MTB asymptomatic** | **CMV seronegative** | **CMV seropositive** | **Toxoplasma seronegative** | **Toxoplasma seropositive** | **HCV seronegative** | **HCV seropositive** |
| --- | --- | --- | --- | --- | --- | --- | --- | --- | --- | --- |
| **Characteristics at plasma sampling** | **N = 2,577** | **N = 263** | **N = 260** | **N = 35** | **N = 424** | **N = 2,360** | **N = 1,415** | **N = 1,374** | **N = 2,069** | **N = 623** |
| Neutralization score | 2 (0, 4) | 1 (0, 3) | 1 (0, 4) | 1 (1, 3) | 1 (0, 4) | 2 (0, 4) | 2 (0, 4) | 2 (0, 4) | 1 (0, 4) | 2 (0, 4) |
| Ethnicity |  |  |  |  |  |  |  |  |  |  |
| White | 2,126 (82%) | 171 (65%) | 196 (75%) | 15 (43%) | 402 (95%) | 1,855 (79%) | 1,170 (83%) | 1,090 (79%) | 1,589 (77%) | 585 (94%) |
| Black | 273 (11%) | 71 (27%) | 46 (18%) | 16 (46%) | 10 (2.4%) | 327 (14%) | 133 (9.4%) | 206 (15%) | 319 (15%) | 12 (1.9%) |
| Other | 178 (6.9%) | 21 (8.0%) | 18 (6.9%) | 4 (11%) | 12 (2.8%) | 178 (7.5%) | 112 (7.9%) | 78 (5.7%) | 161 (7.8%) | 26 (4.2%) |
| Sex [female] | 797 (31%) | 95 (36%) | 78 (30%) | 13 (37%) | 117 (28%) | 759 (32%) | 454 (32%) | 425 (31%) | 619 (30%) | 221 (35%) |
| HIV transmission group |  |  |  |  |  |  |  |  |  |  |
| MSM | 1,072 (42%) | 78 (30%) | 111 (43%) | 12 (34%) | 76 (18%) | 1,055 (45%) | 585 (41%) | 543 (40%) | 1,062 (51%) | 41 (6.6%) |
| HET | 921 (36%) | 122 (46%) | 107 (41%) | 16 (46%) | 169 (40%) | 851 (36%) | 494 (35%) | 532 (39%) | 890 (43%) | 104 (17%) |
| IDU | 495 (19%) | 51 (19%) | 28 (11%) | 5 (14%) | 163 (38%) | 370 (16%) | 286 (20%) | 248 (18%) | 34 (1.6%) | 461 (74%) |
| Other | 89 (3.5%) | 12 (4.6%) | 14 (5.4%) | 2 (5.7%) | 16 (3.8%) | 84 (3.6%) | 50 (3.5%) | 51 (3.7%) | 83 (4.0%) | 17 (2.7%) |
| Age [years] | 37 (32, 44) | 36 (31, 43) | 39 (31, 46) | 35 (31, 44) | 36 (31, 41) | 38 (32, 44) | 36 (31, 42) | 39 (33, 46) | 38 (32, 45) | 36 (32, 40) |
| Cumulative time off ART since HIV infection [years] | 4.2 (3.4, 6.8) | 4.2 (3.7, 6.3) | 3.55 (2.10, 4.09) | 3.65 (2.12, 4.40) | 4.5 (3.5, 8.4) | 4.2 (3.4, 6.5) | 4.2 (3.4, 6.8) | 4.2 (3.3, 6.8) | 4.07 (3.20, 5.55) | 6.0 (3.9, 10.4) |
| Log10 HIV-1 viral load [cps/ml] | 4.33 (3.75, 4.80) | 3.99 (3.40, 4.52) | 4.34 (3.72, 4.76) | 4.23 (3.86, 4.58) | 4.24 (3.63, 4.72) | 4.32 (3.73, 4.80) | 4.27 (3.70, 4.73) | 4.32 (3.74, 4.83) | 4.34 (3.77, 4.81) | 4.22 (3.59, 4.71) |
| CD4 T count [cells/µl] | 393 (297, 532) | 455 (360, 610) | 400 (302, 537) | 421 (339, 605) | 401 (297, 525) | 400 (300, 544) | 400 (299, 550) | 398 (300, 529) | 399 (300, 541) | 398 (296, 531) |
| HIV-1 subtype [non-B] | 639 (25%) | 104 (40%) | 101 (39%) | 21 (60%) | 78 (18%) | 649 (28%) | 342 (24%) | 390 (28%) | 638 (31%) | 75 (12%) |
| HIV-1 diversity [% pol ambiguity] | 1.24 (0.60, 2.23) | 1.24 (0.55, 2.26) | 0.90 (0.41, 1.61) | 1.11 (0.61, 2.21) | 1.27 (0.52, 2.30) | 1.24 (0.61, 2.23) | 1.24 (0.61, 2.25) | 1.27 (0.56, 2.23) | 1.15 (0.54, 2.07) | 1.74 (0.69, 2.69) |
| *^1^* Median (IQR); n (%) | | | | | | | | | | |
