# Supplementary figures and images for: Infection with Mycobacterium tuberculosis alters the antibody response to HIV-1
Source: PLoS Pathog. 2025 Aug 13;21(8):e1013350. doi: 10.1371/journal.ppat.1013350 (PMC12370194; doi:10.1371/journal.ppat.1013350)

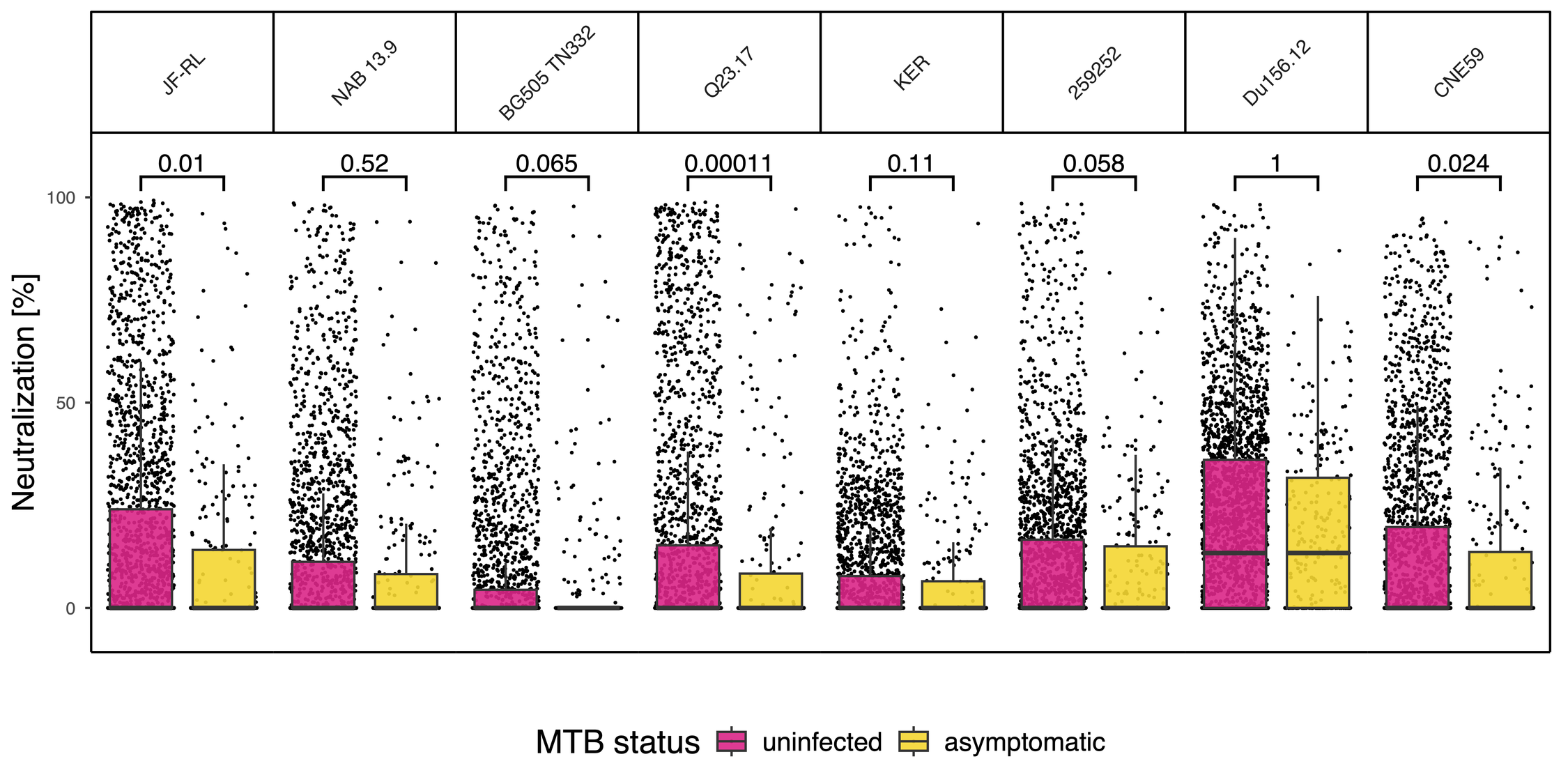

Supplement: S1 Fig — Distributions of plasma neutralization ranging from 0 to 100, whereas 100 indicates full neutralization of the respective HIV-1 strain. MTB status was defined as i) MTB uninfected, defined through presence of a negative tuberculin skin test (TST) or interferon gamma release assay (IGRA) any time before or 1 year post baseline. ii) Asymptomatic MTB infection, defined either through a positive TST or IGRA any time before or 1 year post baseline, or as progression to active TB > 180 days post baseline P values are derived from a tobit model additional adjusted for demographic characteristics and HIV-1 disease specific parameters. (TIF) [file ppat.1013350.s004.tif]

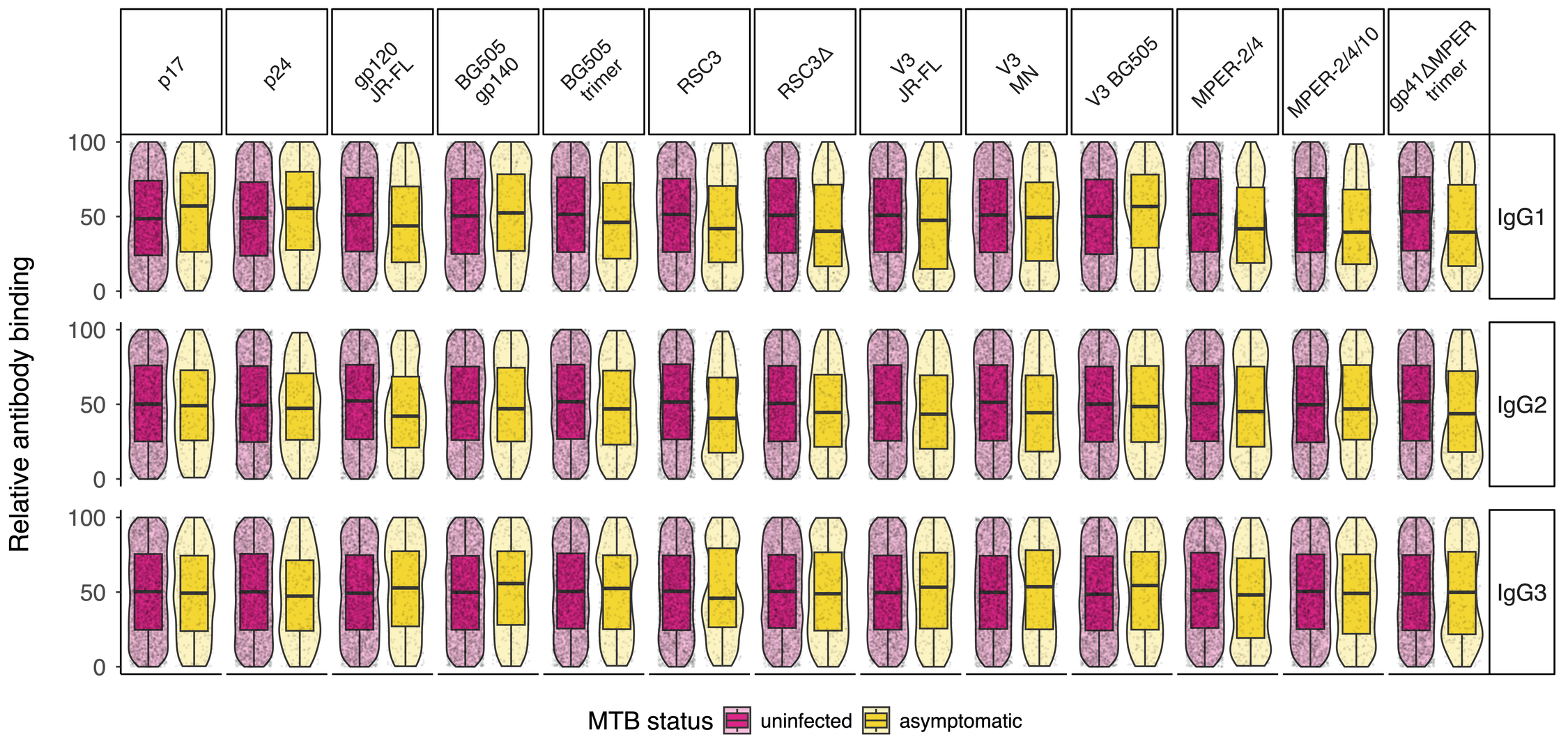

Supplement: S2 Fig — Ab binding was measured as mean fluorescent intensity (MFI) transformed to a relative binding ranging from 0 to 100, whereas 100 indicates strongest binding. MTB status was defined as i) MTB uninfected, defined through presence of a negative tuberculin skin test (TST) or interferon gamma release assay (IGRA) any time before or 1 year post baseline. ii) Asymptomatic MTB infection, defined either through a positive TST or IGRA any time before or 1 year post baseline, or as progression to active TB > 180 days post baseline. (TIF) [file ppat.1013350.s005.tif]

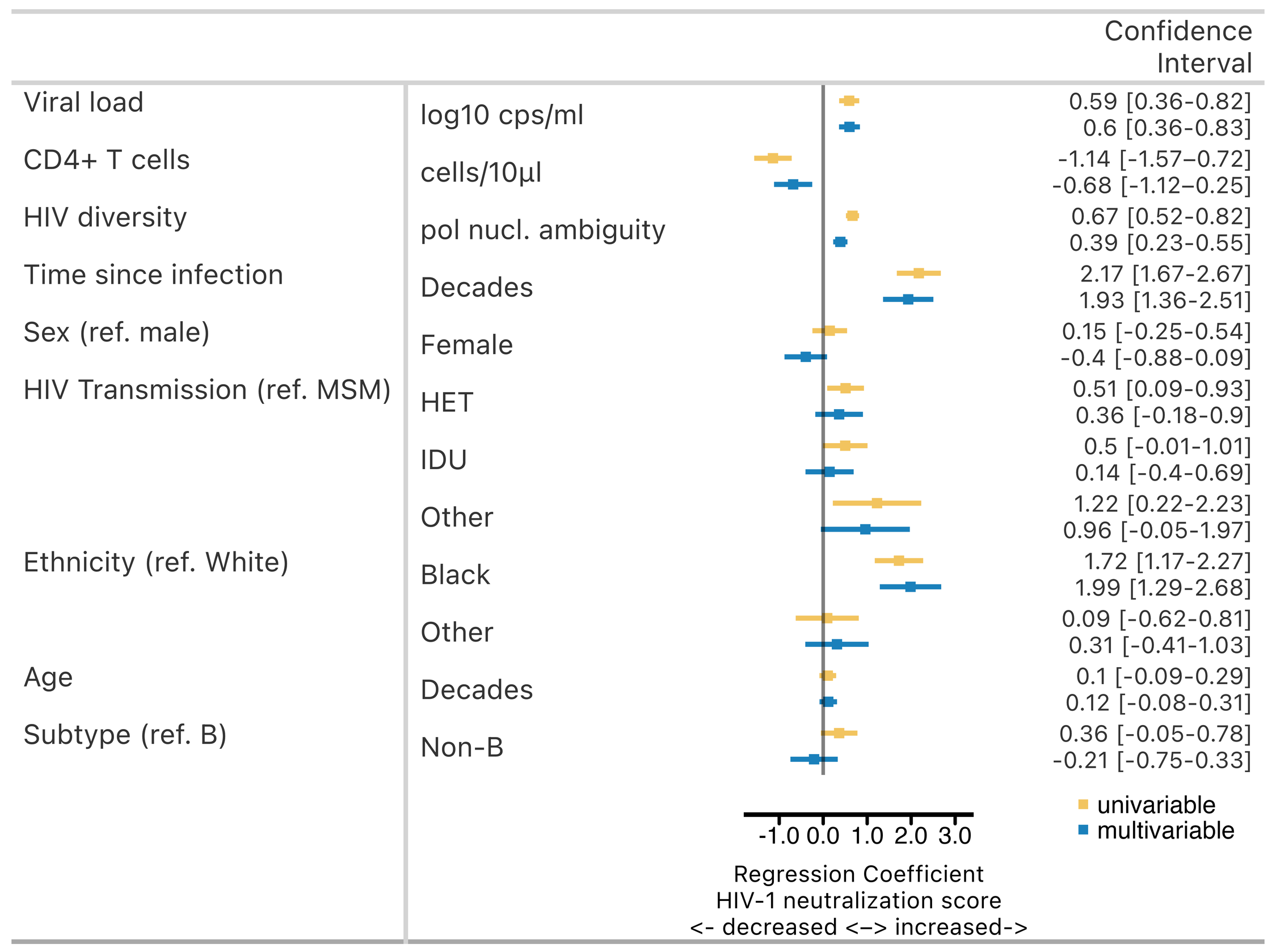

Supplement: S3 Fig — Neutralization score was determined from observed plasma neutralization against eight HIV-1 strains from five subtypes, ranging from 0 to 100% neutralization. For each strain a score was calculated, 3 when neutralization >80%, 2 when neutralization 50% to <80%, 1 when neutralization 20% to <50%, and 0 when neutralization <20%. The sum of the individual virus scores were then summarized as the neutralization score (ranging from 0 to maximum 24). The effect estimates were determined with a tobit regression. (TIF) [file ppat.1013350.s006.tif]

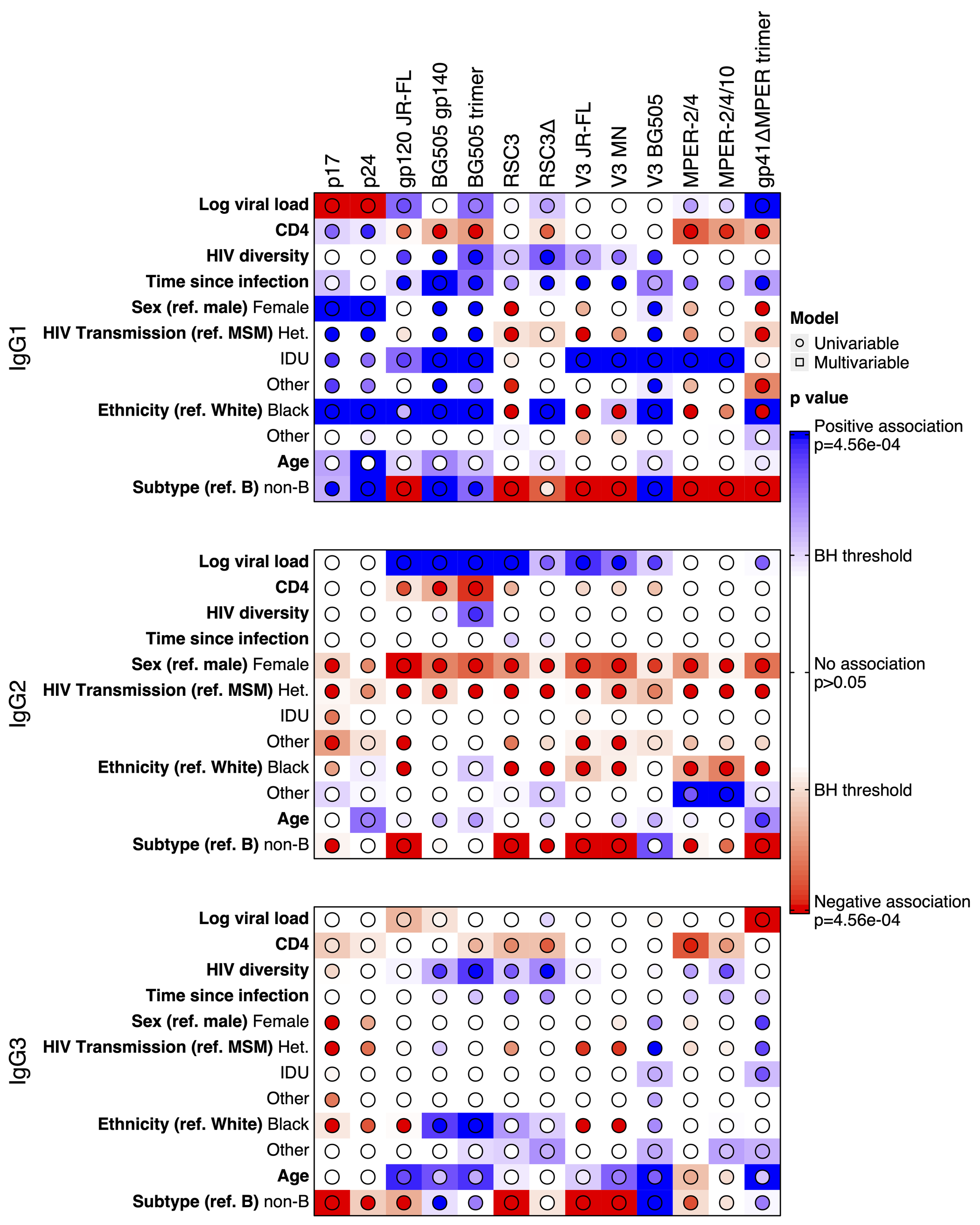

Supplement: S4 Fig — Ab binding was measured as mean fluorescent intensity (MFI) transformed to a relative binding ranging from 0 to 100, whereas 100 indicates strongest binding. Inner circles describe univariable results and brackets describe multivariable results, adjusted for all other demographic characteristics and HIV-1 disease specific parameters. The p values and effect estimates were determined with a linear regression. (TIF) [file ppat.1013350.s007.tif]

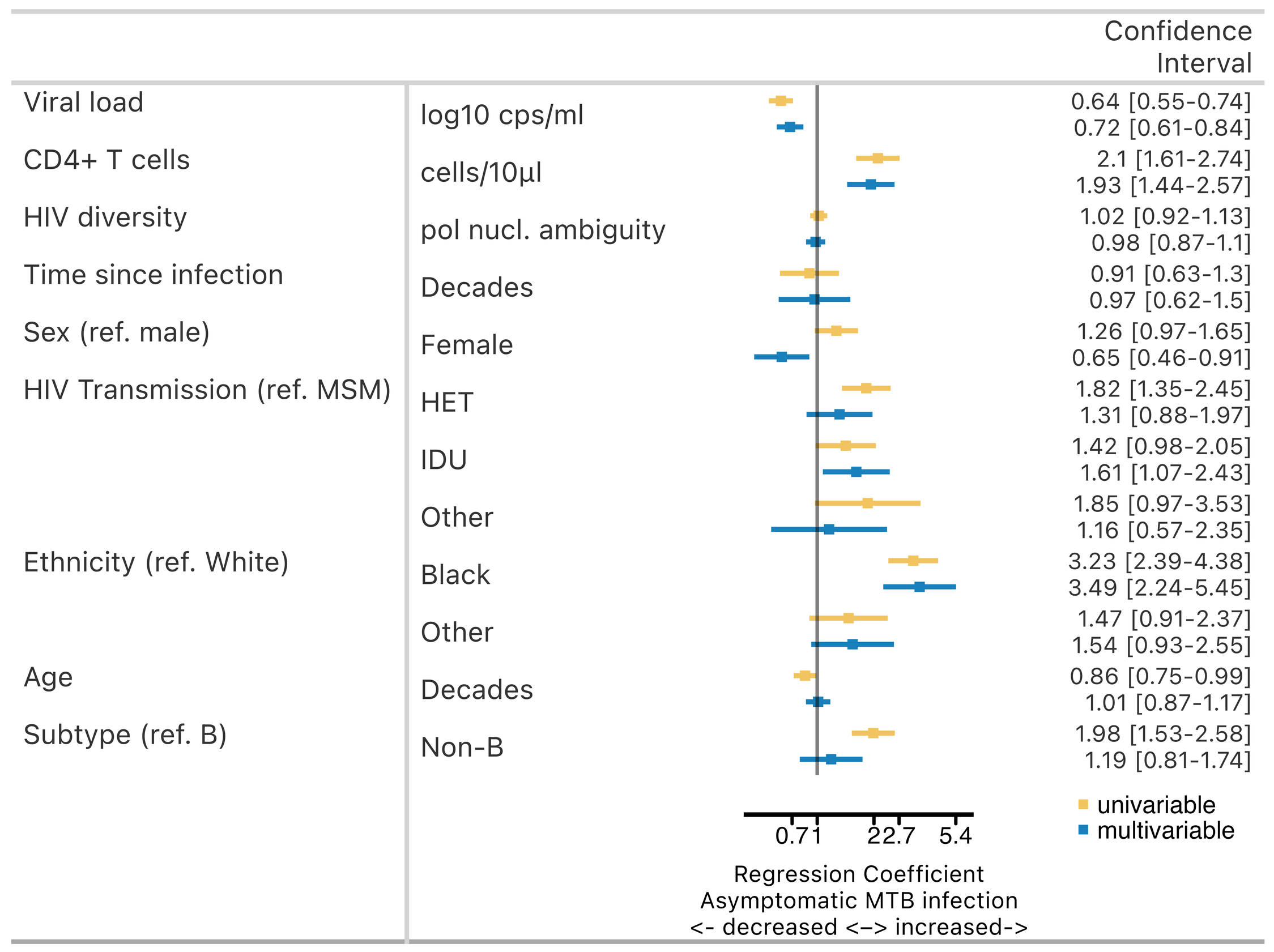

Supplement: S5 Fig — MTB status was defined as i) MTB uninfected, defined through presence of a negative tuberculin skin test (TST) or interferon gamma release assay (IGRA) any time before or 1 year post baseline. ii) Asymptomatic MTB infection, defined either through a positive TST or IGRA any time before or 1 year post baseline, or as progression to active TB > 180 days post baseline. The effect estimates were determined with a logistic regression. (TIF) [file ppat.1013350.s008.tif]

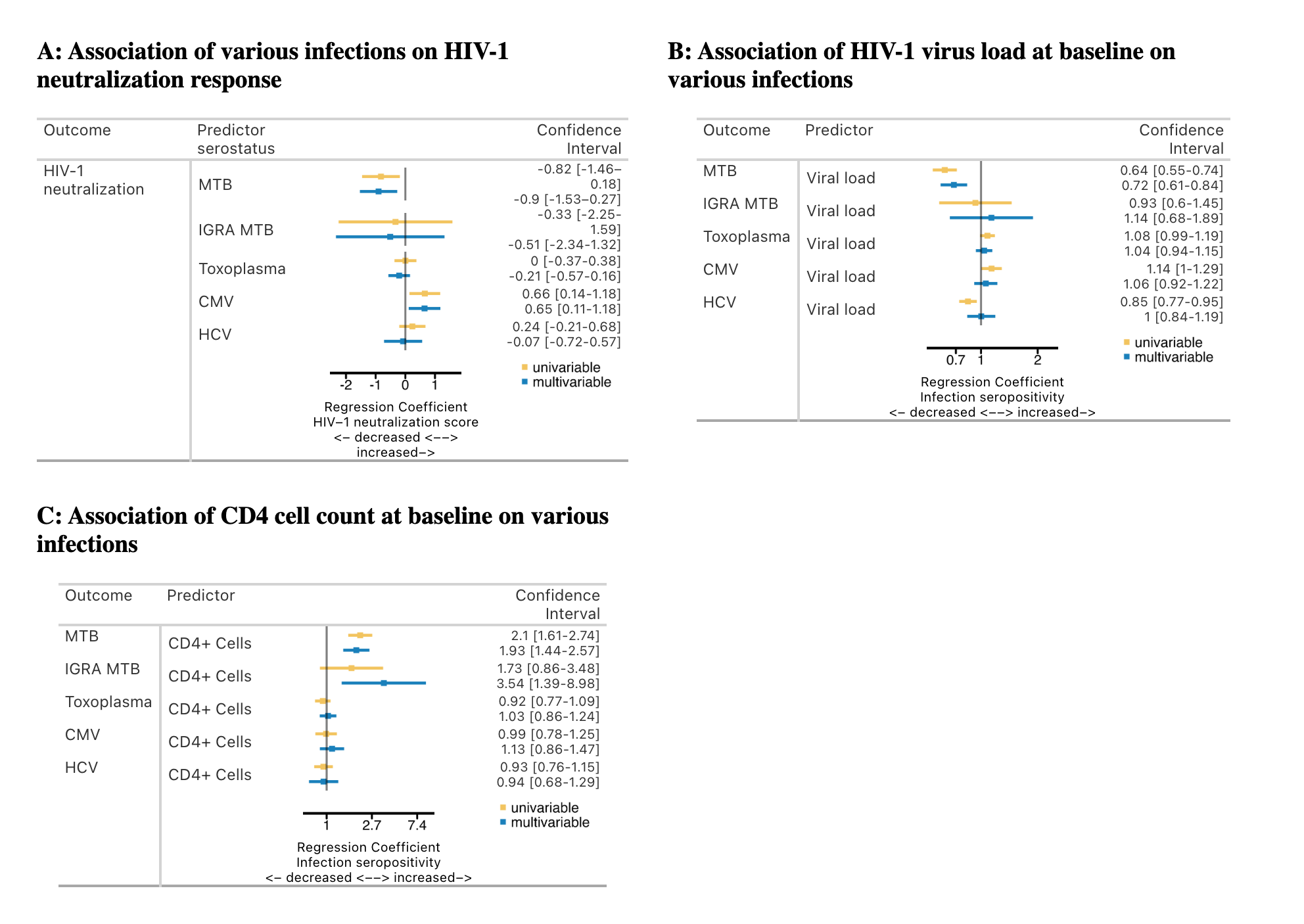

Supplement: S6 Fig — A: HIV-1 neutralization score and associations with demographic characteristics and HIV-1 disease specific parameters. The effect estimates were determined with a tobit regression. B/C: Serostatus of MTB, Toxoplasma gondii, CMV, and HCV and associations with HIV RNA load and CD4 + T cell counts. MTB serostatus status was defined as i) MTB uninfected, defined through presence of a negative tuberculin skin test (TST) or interferon gamma release assay (IGRA) any time before or 1 year post baseline. ii) Asymptomatic MTB infection, defined either through a positive TST or IGRA any time before or 1 year post baseline, or as progression to active TB > 180 days post baseline. CMV, Toxoplasma gondii, and HCV serostatus were defined based on IgG antibody tests any time before or 1 year post baseline. The effect estimates were determined with a logistic regression. (TIF) [file ppat.1013350.s009.tif]

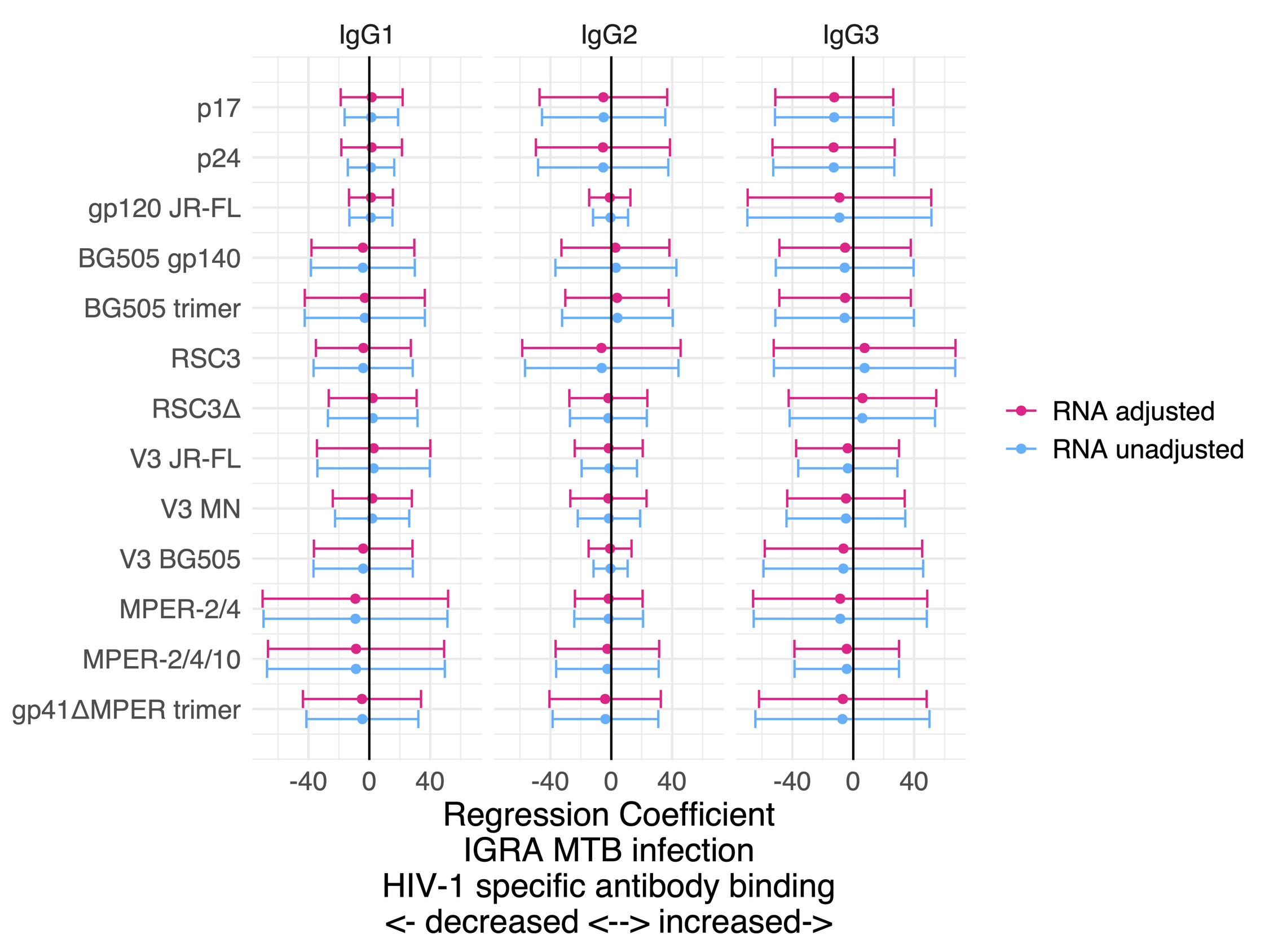

Supplement: S7 Fig — Adjusted for demographic characteristics and HIV-1 disease specific parameters. RNA unadjusted corresponds to regression models not adjusted for HIV-1 RNA viral load. The effect estimates were determined with a linear regression and the confidence intervals were adjusted based on the p values adjusted for multiple testing using the Benjamini-Hochberg procedure. (TIF) [file ppat.1013350.s010.tif]

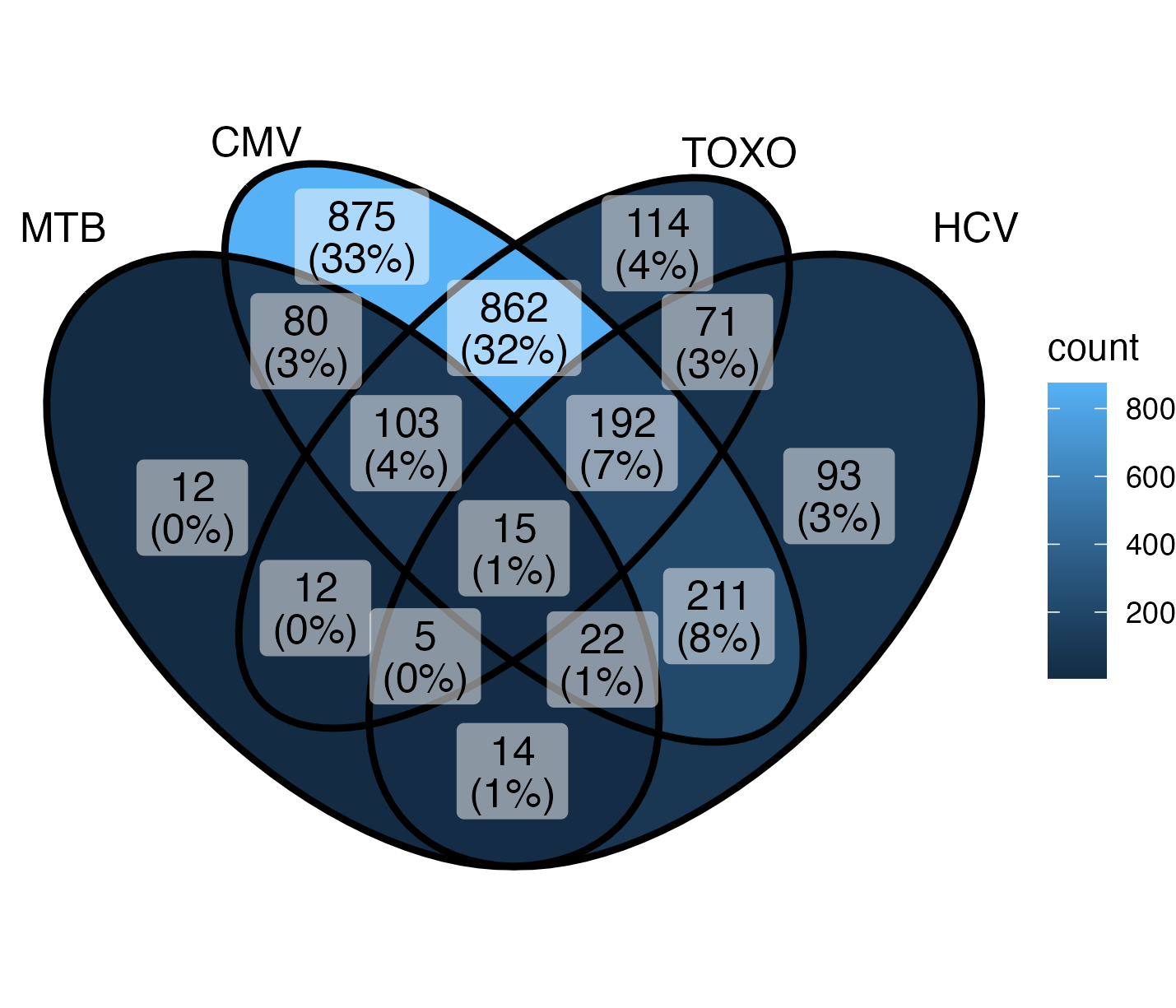

Supplement: S8 Fig — Depicted is the overlap of seropositive tests in the study population against MTB (Mycobacterium tuberculosis), CMV (Cytomegalovirus), TOXO (Toxoplasma gondii), and HCV (Hepatitis C virus). (TIF) [file ppat.1013350.s011.tif]

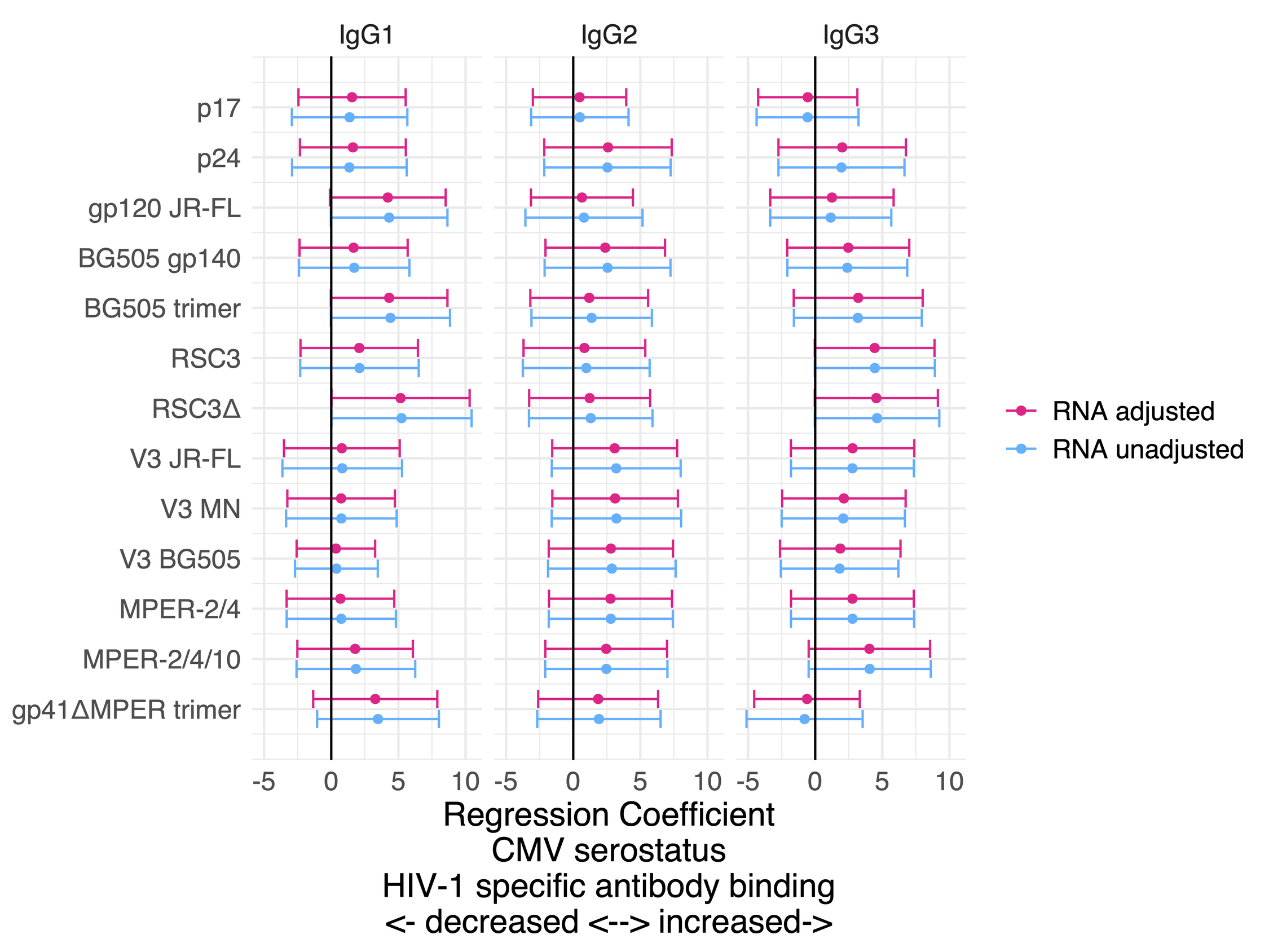

Supplement: S9 Fig — Adjusted for demographic characteristics and HIV-1 disease specific parameters. RNA unadjusted corresponds to regression models not adjusted for HIV-1 RNA viral load. The effect estimates were determined with a linear regression and the confidence intervals were adjusted based on the p values adjusted for multiple testing using the Benjamini-Hochberg procedure. (TIF) [file ppat.1013350.s012.tif]

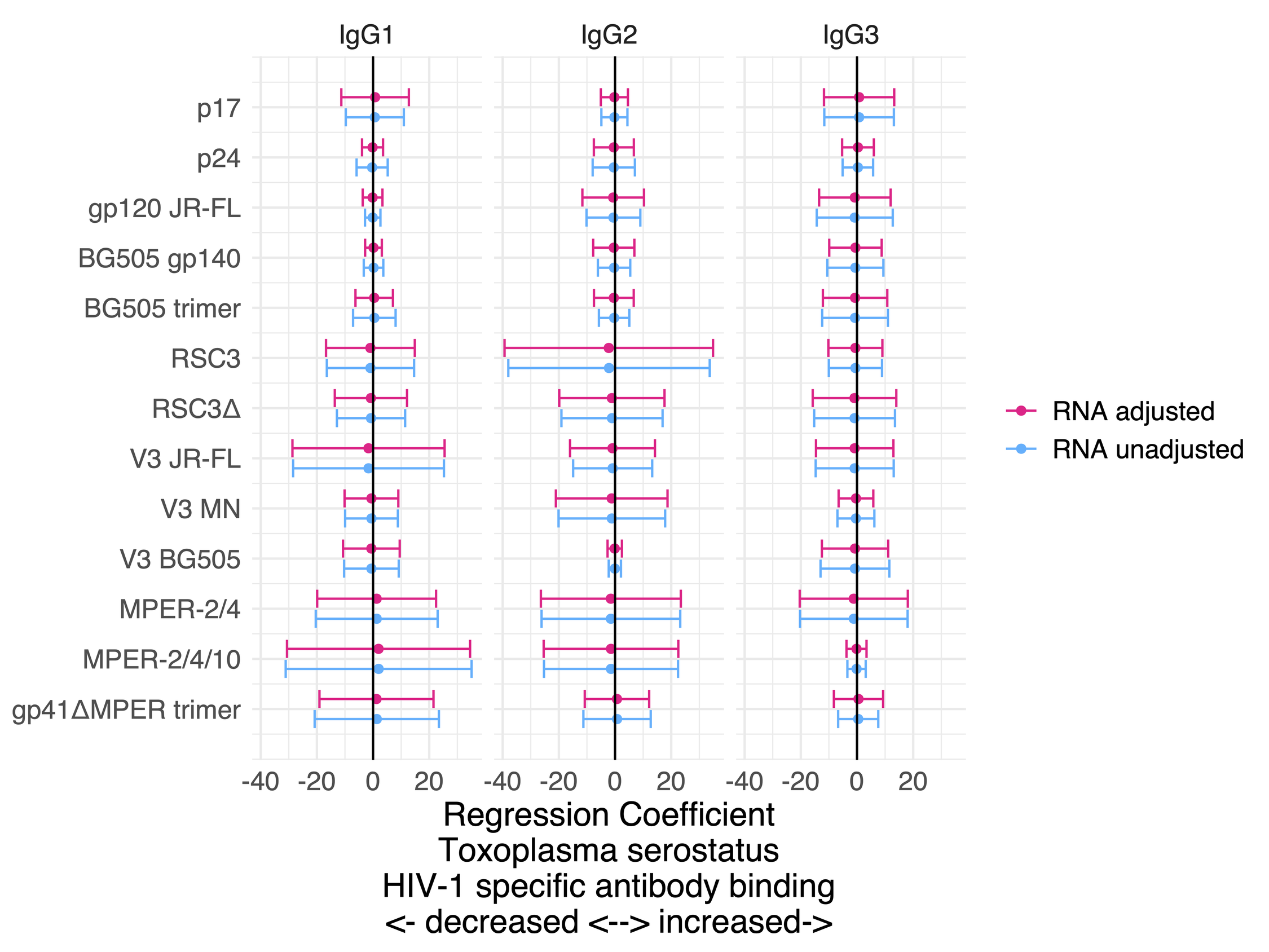

Supplement: S10 Fig — Adjusted for demographic characteristics and HIV-1 disease specific parameters. RNA unadjusted corresponds to regression models not adjusted for HIV-1 RNA viral load. The effect estimates were determined with a linear regression and the confidence intervals were adjusted based on the p values adjusted for multiple testing using the Benjamini-Hochberg procedure. (TIF) [file ppat.1013350.s013.tif]

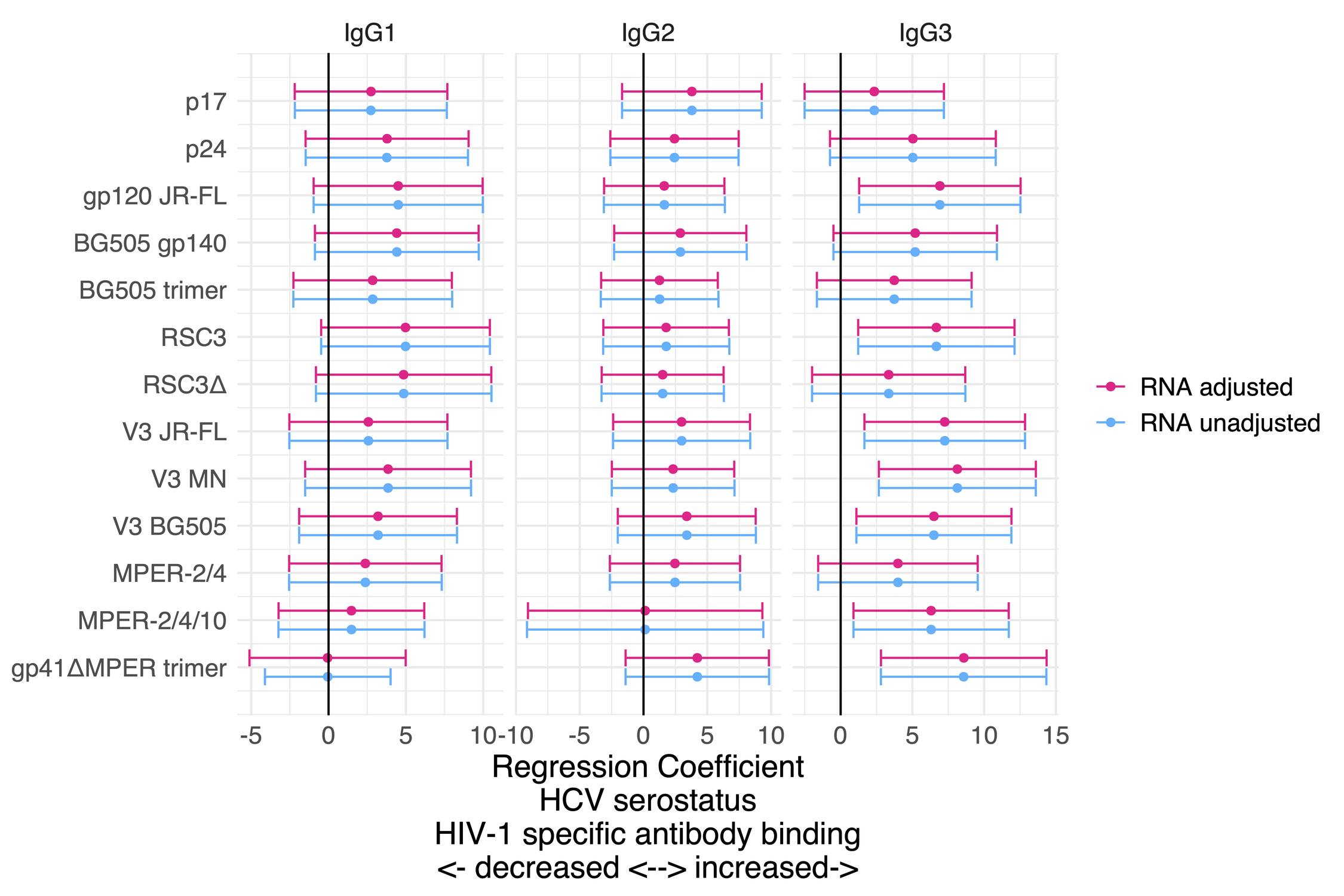

Supplement: S11 Fig — Adjusted for demographic characteristics and HIV-1 disease specific parameters. RNA unadjusted corresponds to regression models not adjusted for HIV-1 RNA viral load. The effect estimates were determined with a linear regression and the confidence intervals were adjusted based on the p values adjusted for multiple testing using the Benjamini-Hochberg procedure. (TIF) [file ppat.1013350.s014.tif]

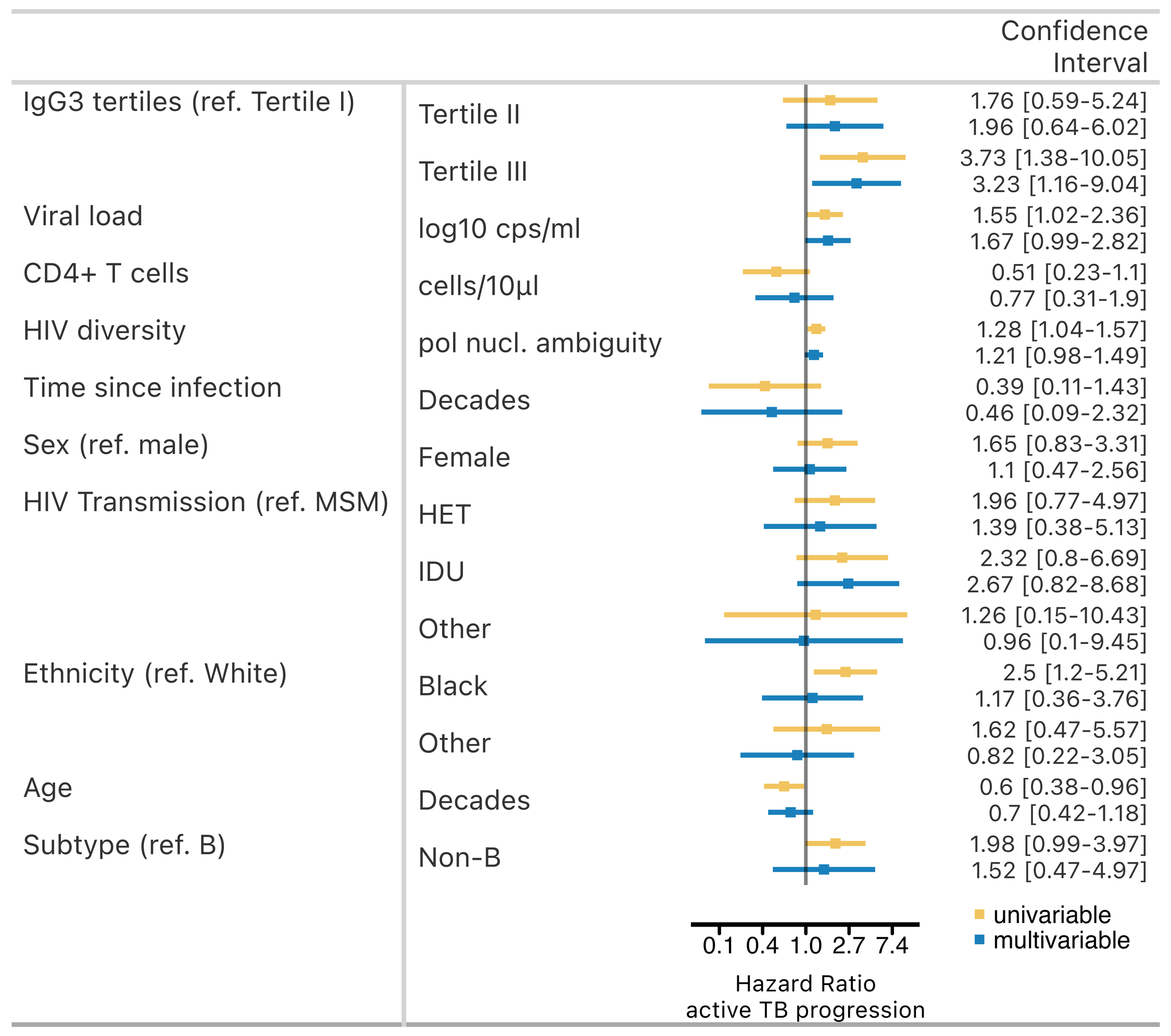

Supplement: S12 Fig — Adjusted comparison with demographic characteristics and HIV-1 disease specific parameters. Progression to active TB was defined as diagnosis of active TB > 180 days post baseline. Comparison group was defined through a positive TST or IGRA any time before or 1 year post baseline. The effect estimates were determined with a cox proportional hazard regression. (TIF) [file ppat.1013350.s015.tif]

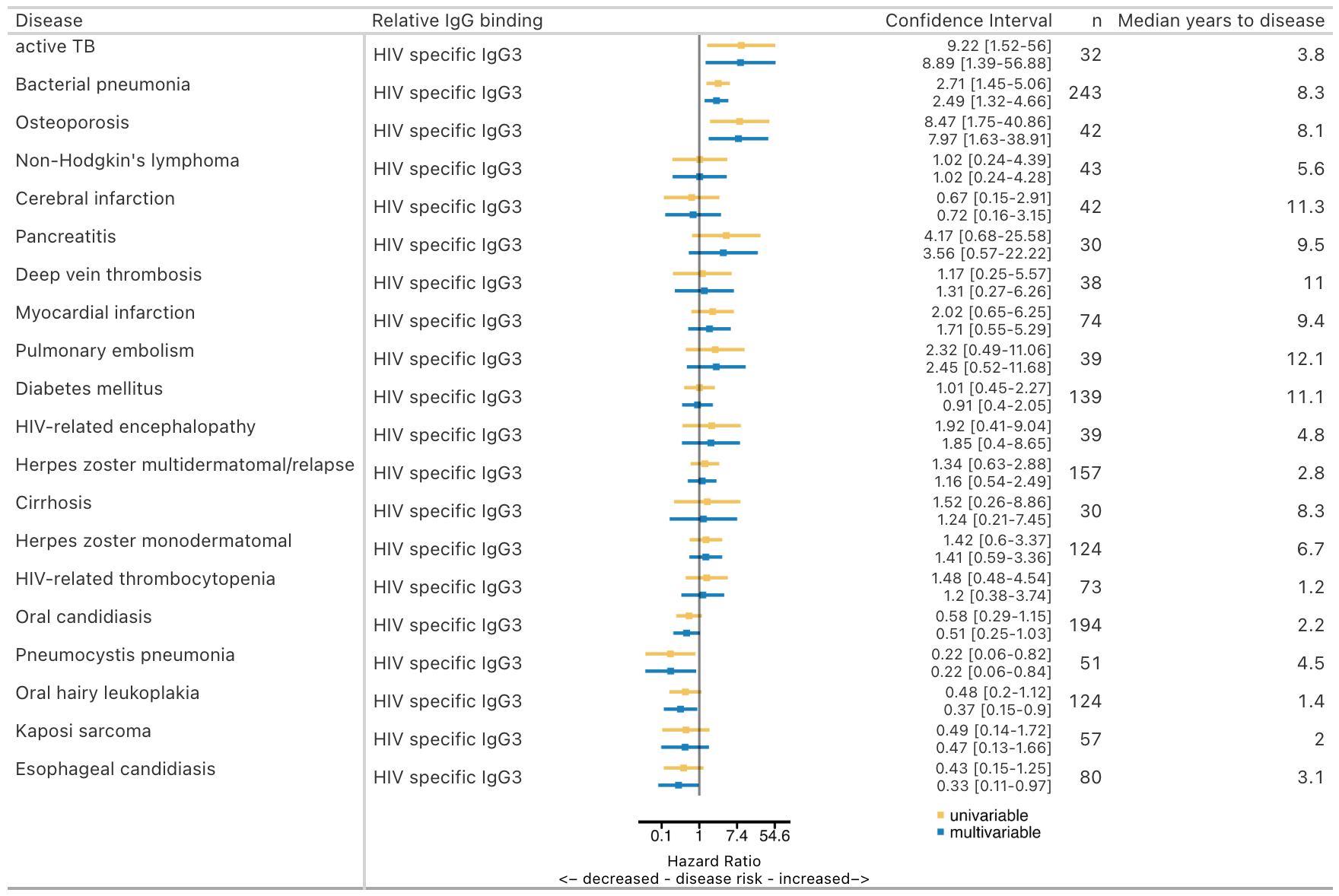

Supplement: S13 Fig — Adjusted comparison with demographic characteristics and HIV-1 disease specific parameters. Progression to opportunistic infection or non-communicable disease was defined as diagnosis of the respective disease >180 days post baseline. The effect estimates were determined with a cox proportional hazard regression. (TIF) [file ppat.1013350.s016.tif]
