# Supplementary material for: Infection with Mycobacterium tuberculosis alters the antibody response to HIV-1
Source: PLoS Pathog. 2025 Aug 13;21(8):e1013350. doi: 10.1371/journal.ppat.1013350 (PMC12370194; doi:10.1371/journal.ppat.1013350)
Supplement: Attachment — Submitted filename: Response_to_Reviewers.docx [file ppat.1013350.s018.docx]

**Response to Reviewers**

**Editor’s comments:**

Thank you for submitting your manuscript to PLOS Pathogens. After careful consideration, we feel that it has merit but does not fully meet PLOS Pathogens's publication criteria as it currently stands. Therefore, we invite you to submit a revised version of

the manuscript that addresses the points raised during the review process.

**Reply:** We like to thank the editor and the reviewers for considering our work and provide constructive comments for improvements. Please find below our detailed point-by-point reply to the comments.

**Reviewers' Comments:**

**Part I - Summary**

**Reviewer #1:**

This article focuses on the interaction between TB/HIV co-infection and the specific B cell response to HIV. This retrospective study analysed 263 HIV-infected individuals (231 with latent tuberculosis and 32 with active tuberculosis during follow-up). The authors tested the levels of HIV antibodies in the two groups and then compared them with those infected with HIV without TB infection. The main conclusion is that active or asymptomatic TB infection alters the development of HIV-specific antibodies.
Strengths
- The study includes a well-established, well-followed cohort
- The tests described appear to be standardised in the laboratory.
Weaknesses
- Selection of individuals to be analysed
- Control groups to validate the analysis

**Reviewer #2:**

This brief manuscript from Zeeb, et al. describes a retrospective study of plasma from PWH with or without Mtb infection to investigate antibodies binding/neutralization to HIV. The data suggests that Mtb infection, whether subclinical or symptomatic, can influence antibody responses to HIV. While many studies have looked at Ab responses to HIV or to Mtb, few have looked at the effect of TB status on anti-HIV responses. Published data from the SHCS offered a unique opportunity to do so. Importantly, only data from PWH off ART were included. Appropriate statistical tests were applied. The analyses revealed that Mtb coinfection was associated with reduced HIV-1 neutralization. Interestingly, PWH who went on develop active TB exhibited higher IgG3 even though prior studies suggest that IgG3 protects from TB disease. This inconsistency is adequately discussed and the limitations of this analysis are stated.
In summary, this retrospective study of the influence of Mtb on anti-HIV responses is novel. It is also very simplistic as Ab glycosylation or functional roles are not considered, presumably because these data do not exist from the sample set. The report is well-written, the analyses are sufficient for the stated purpose, and the conclusions are not over-drawn. However, the limited scope of the serological data likewise limits the significance of this contribution.

**Reviewer #3:**

This manuscript by Zeeb et al builds on a previous publication from this group showing that in the Swiss HIV Cohort study, showing a surprising association between LTBI or Mtb-uninfected status and lower HIV set point viral load (SPVL) when compared to PWH with Active TB. They also reported fewer opportunistic infections overall in HIV/Mtb coinfected patients with LTBI. Here they attempt to investigate (in a retrospective study) the potential immunological underpinnings of the association that they previously reported.

Using the SHCS, they studied PWH with and without TST+/IGRA+ tests at baseline and assessed HIV-1-specific plasma binding/neutralizing responses in individuals who remained asymptomatic and in those who developed active TB. They report that PWH with asymptomatic Mtb had reduced HIV-1 Ab levels and neutralization capacities compared to PWH with no Mtb while PWH who developed active TB showed a preferential shift to IgG3 Abs, showing that PWH in these 2 categories diverge in their Ab responses to HIV.

Overall the findings are interesting and the study is well designed. Combined with the results from their previous study in PLos Biology, their observations raise some interesting issues regarding how prior HIV infection might potentially influence Mtb infection outcomes. However, while these findings report an association between asymptomatic MTB infection and reduced binding of HIV-1 Abs antibodies to specific HIV antigens and, reduced neutralization capacity, the underlying correlates and mechanisms remain unclear.

**Reply:**

We sincerely thank the reviewers for their constructive comments. In response, we have substantially revised the manuscript to address these issues and strengthen the scientific foundation of our findings.

**We now show that Mycobacterium tuberculosis (MTB) co-infection in people with HIV (PWH) uniquely disrupts HIV-specific humoral immunity**, a finding that has not been previously demonstrated in this depth.

This includes:

- **New analyses of serostatus of other common co-infections** (CMV, HCV, *Toxoplasma gondii*), revealing that none replicate the suppressive effect of MTB on HIV-1 neutralization or HIV-1 Ab binding. Presented in new Supplementary Figures (6–11) and Supplementary Table 3, these results emphasize that the immunomodulatory effects are specific to MTB.
- **New analyses of other opportunistic infections and non-communicable diseases** shows that elevated HIV-specific IgG3 responses precede not only active TB but also bacterial pneumonia and osteoporosis (Supplementary Fig. 13), indicating broader predictive and possibly mechanistic implications of IgG3 dynamics in PWH.
- **Clarified rationale for cohort selection**, including new sensitivity analyses restricted to IGRA-tested individuals (Supplementary Table 3 and Fig. 6–7) to address diagnostic specificity.

These additions directly address the reviewer’s concerns on cohort selection and lack of controls. Moreover, the **revised discussion now integrates current literature on humoral responses in TB/HIV co-infection**, adding biological context and citing recent transcriptomic and antibody studies.

Taken together, we strongly believe that these revisions strengthen the manuscript’s novelty and validity. We are confident the revised version presents a clearer, more comprehensive, and more scientifically rigorous narrative of how MTB co-infection shapes HIV-specific antibody responses.

**Part II – Major Issues: Key Experiments Required for Acceptance**

**Reviewer #1:**

1. General comments
   In terms of B cell responses, the interaction between TB infection and HIV is very complex. Both monoinfections induce changes in circulating B cells, B cell repertoire and levels of specific and non-specific immunoglobulins. Both TB and HIV induce polyclonal activation, and this polyclonal activation can alter the levels of other specific antibodies. The changes in circulating B cells and B cell repertoire are described in latent and active tuberculosis. IgG3 (specific or non-specific) are not only described in tuberculosis but in other intracellular infections. Thus, the question is whether the results described here are specific to tuberculosis infection or observed in other intracellular co-infections. Furthermore, are these only changes induced by tuberculosis infection? Thus, results presented here do not allow us to distinguish between tuberculosis mono-infection and HIV infection.

**Reply:** We appreciate the reviewer’s insightful comments regarding the complexity of B cell responses in TB and HIV infection, and the challenge of disentangling effects due to mono- versus co-infection. Indeed, as highlighted, both HIV and TB can independently induce polyclonal B cell activation, alter the circulating B cell repertoire, and impact immunoglobulin levels, including IgG3. However, we would like to emphasize that our findings are fully consistent with this complex immunological landscape, and in fact strengthen the central hypothesis of our study—that TB co-infection contributes to shaping of the humoral immune response in individuals with HIV.

As suggested by the reviewer, we extended our analysis to additional pathogens using available serostatus data for other intracellular pathogens, including CMV, *Toxoplasma gondii*, and HCV in our cohort. We show the overlap between the different positive serostatus in a Venn diagram in Supplementary Fig. 8. The population characteristics at baseline stratified by individual infection serostatus are now shown in Supplementary Table 3. Notably, *Toxoplasma gondii* and HCV seropositivity did not associate with altered HIV-specific neutralization (shown now in Supplementary Fig. 6A). CMV exhibited an opposite pattern to MTB, with increased HIV-1 neutralization. Additionally, CMV and *Toxoplasma gondii* did not associate with altered HIV-specific binding (Supplementary Fig. 9,10). HCV exhibited an opposite pattern to MTB, with increased IgG3 binding (Supplementary Fig. 11). Both the CMV and HCV signals highlight the distinct immunomodulatory signature of TB co-infection on the HIV-antibody response. Importantly, unlike MTB, seropositivity for none of these pathogens was associated with HIV disease progression markers (HIV RNA levels or CD4+ T cell counts), underscoring the specificity of the TB-associated immune alterations (see Supplementary Fig. 6C/D).

While we acknowledge that the current dataset does not allow us to study MTB mono-infection, the strength of our approach lies in the comparison across multiple co-infections within the same HIV-infected cohort. These data support our overarching conclusion: TB co-infection elicits a unique humoral immune signature, distinguishable from both HIV mono-infection and other common co-infections.

We accordingly made the following changes in the manuscript:

Line 206ff in the methods: “We assessed if association patterns with asymptomatic MTB infection are also observed with seropositivity to other infections among PWH with known MTB status. We considered the serostatus of CMV, *Toxoplasma gondii*, and HCV (based on IgG antibody tests any time before or 1 year post baseline) and repeated the analyses on HIV-1 neutralization score and HIV-1 Ab binding.”

Line 311ff in the results: “We determined if seropositivity to other infections exhibits similar patterns on the HIV-1 neutralization and HIV-1 Ab response. We assessed the association with serostatus of *Toxoplasma gondii* (n=1374), CMV (n=2360), and HCV (n=623). The demographics are shown in S1 Table and the overlap between infections in S8 Fig. None of those pathogens reduced the HIV-1 neutralization like asymptomatic MTB, only CMV was associated with an 0.69 increase (CI = 0.11, 1.18) of neutralization (S6A Fig). Similar, none of those pathogens were associated with reduced HIV-1 Ab reactivity (S9-11 Fig). However, we observed increased IgG3 reactivity, against a range of antigens, associated with HCV seropositivity (S11 Fig). Importantly, unlike MTB, seropositivity for none of these pathogens was associated with HIV-1 disease progression markers (HIV RNA levels or CD4+ T cell counts), underscoring the specificity of the TB-associated immune alterations (see S6C/D Fig).”

Since in the original manuscript we not only reported the impact of MTB infection on HIV-1 specific neutralization and binding reactivity, but also, conversely, the association of HIV-1 specific IgG3 reactivity with later progression to active TB, we performed a second analysis to also validate those results. In this analysis, we evaluated a broad range of opportunistic infections and non-communicable conditions (n = 19) for associations with the HIV-1 IgG3 response. Across these 19 diverse outcomes, two, namely bacterial pneumonia and osteoporosis mirrored the IgG3 pattern observed among individuals who later developed TB (see Supplementary Fig. 13). Meaning high HIV IgG3 indicates also an elevated risk for osteoporosis and bacterial pneumonia in the future.

The fact that next to MTB other bacteria causing pulmonary infections lead to shifts in IgG1/IgG3 responses is very intriguing suggesting a shared mechanism of immune perturbation. This aligns with the known role of IgG3 in responding to protein antigens and its involvement in defense against bacterial pathogens (Damelang, Trends in Immunology, 2019, doi.org/10.1016/j.it.2019.01.005).

Regarding osteoporosis, the association with elevated anti-HIV-1 IgG3 levels is novel and, to our knowledge, has not been previously reported. While the underlying mechanisms remain to be fully elucidated, recent studies have shed light on the complex interplay between IgGs and bone metabolism. For instance, Nurkkala-Karlsson et al. (Scientific Reports, 2024, doi.org/10.1038/s41598-024-56192-1) report that, in 546 elderly men of the Osteoporotic Fracture in Men cohort Study in Sweden, overall IgG may lead to bone loss. Additionally, several *in vitro* studies showed that the Fc region of IgG antibodies promotes osteoclastogenesis and bone loss by binding to the Fcγ receptors of osteoclasts (Sehic, Scand. J. Immunol, 2021, doi.org/10.1111/sji.13009; Harre, Nat. Commun., 2015; Kamohara, Int. Immunol., 2020, doi.org/10.1093/intimm/dxz063).These findings suggest that elevated overall IgG levels may influence bone remodeling processes, potentially contributing to osteoporosis development.

In contrast, conditions such as esophageal candidiasis, pneumocystis pneumonia, and oral hairy leukoplakia exhibited lower pre-onset IgG3 levels (see Fig. 6), reinforcing the notion that elevated IgG3 is not a generalized feature of immune activation or comorbidity but may be a distinctive hallmark of specific bacterial infections and bone-related pathologies in the context of HIV.

We accordingly made the following changes in the manuscript:

Line 211ff in the methods: ”We assessed if later progression to 19 other opportunistic infections and non-communicable conditions, besides active TB, is associated with IgG responses. We considered opportunistic infections and non-communicable conditions with n >30 among the PWH with known MTB status: Oral candidiasis, Herpes zoster multidermatomal/relapse, Oral hairy leukoplakia, Esophageal candidiasis, Kaposi sarcoma, Non-Hodgkin's lymphoma, HIV-related encephalopathy, HIV-related thrombocytopenia, Pneumocystis pneumonia, Myocardial infarction, Bacterial pneumonia, Cerebral infarction, Cirrhosis, Diabetes mellitus, Herpes zoster monodermatomal, Osteoporosis, Pancreatitis, Deep vein thrombosis, and Pulmonary embolism.”

Line 350ff in the results: “In a sensitivity analysis assessing subsequent disease progression among all PWH, we identified similar associations between elevated HIV-specific IgG3 levels and the later development of bacterial pneumonia (HR 2.49, 95% CI: 1.32–4.66) and osteoporosis (HR 7.97, 95% CI: 1.63–38.91) (S13 Fig), suggesting that the IgG3 signature may reflect a broader shift in immune homeostasis.”

Line 413ff in the discussion: “PWH with elevated anti-HIV-1 IgG3 levels were more likely to subsequently develop active TB, bacterial pneumonia, or osteoporosis, suggesting a broader humoral immune response. Notably, this IgG3 shift was detectable well before disease onset—with median lead times of 3.8 years for TB, and 8.3 and 8.1 years for pneumonia and osteoporosis, respectively.”

**Reviewer #2:**

None

**Reviewer #3:**

1. Admittedly, as acknowledged by the authors, establishing causal links is challenging, however since longitudinal samples appear to be available through the SHCS, there is potential to generate these data to strengthen the study. Other data that could strengthen the study is to provide evidence of immune perturbations by analyzing plasma for inflammatory mediators that might correlate with outcomes.

**Reply:** We thank the reviewer for this thoughtful and constructive suggestion. We fully agree that integrating longitudinal and functional data is crucial to unraveling causal links and better understanding the immunological consequences of MTB infection in PWH.

In fact, we have already taken important steps in this direction. As detailed in our recent study (Tepekule, iScience, 2024, doi.org/10.1016/j.isci.2024.111228), we performed a transcriptomic analysis of peripheral blood mononuclear cells (PBMCs) from well-characterized individuals within the Swiss HIV Cohort Study (SHCS). This study demonstrated that MTB infection, even in the absence of active disease, induces marked and consistent transcriptional perturbations that were independent of HIV viremia. Notably, we identified sustained activation of inflammatory pathways—particularly involving TNF-α, IL-6, and KRAS signaling—that were inversely associated with HIV viral load. These transcriptomic changes were not due to differences in PBMC subpopulations, as confirmed by flow cytometry, and were consistent across individuals with and without HIV suppression. Furthermore, we developed a “conserved transcript” signature score, integrating genes consistently altered by MTB infection, which also correlated with lower HIV viral load. Interestingly, this protective transcriptional profile was diminished in individuals who progressed to active TB, providing a potential mechanistic link between immune activation states and retroviral control.

In addition, we are currently undertaking a comprehensive proteomic profiling study using longitudinal plasma samples from the SHCS. This effort aims to quantify circulating inflammatory mediators and delineate immune signatures that may predict or mediate disease outcomes. The scale and depth of this proteomic screening will allow us to explore the molecular interface between MTB exposure and systemic immune activation in unprecedented detail.

In summary, our current and forthcoming investigations are strongly aligned with the reviewer’s suggestion. The transcriptional changes we observed offer initial evidence of MTB-induced immune perturbations, and the proteomic analyses underway will provide a crucial next layer of insight.

However, due to the scope and timeline of the current manuscript, this ongoing proteomic work is not yet ready for inclusion. We believe that integrating these data will significantly enrich future publications and allow for more mechanistic insights into the observed associations.

2. While IGRA+/TST+ data are reported the numbers who were IGRA+ is not outlined. Since TST does not distinguish between BCG and Mtb, it is important to show analysis of the IGRA+ results separately in order to interpret the impact of asymptomatic Mtb infection.
   Overall, the data presented here are intriguing but fall short in the absence of providing additional data to provide insight into the biological implications behind the observations.

**Reply:** We thank the reviewer for highlighting the importance of distinguishing IGRA+ from TST+ individuals, especially given the known limitations of TST in BCG-vaccinated populations. In response, we conducted a sub-analysis restricted to individuals with either positive (n=35) or negative (n=260) IGRA results. While the smaller sample size limits statistical power, the observed trends — particularly after multivariable adjustment — were consistent with the findings, with the exception of HIV-1 binding which showed no trends, when considering both IGRA+ from TST+, though they did not reach statistical significance. Notably, a positive association with CD4+ T cell count was observed. (see Fig.1D)

We would also like to provide further context on IGRA use within the Swiss HIV Cohort Study (SHCS). IGRA testing was introduced in the early 2000s, around the same time that modern, highly effective antiretroviral therapy (ART) became widely available. However, the antibody measurements in our study were mostly taken earlier in the HIV pandemic, when IGRA was not yet in routine use. At that time, testing for asymptomatic MTB infection in people with HIV was primarily done using the TST. Since IGRA was introduced later and used mostly in individuals who were already on ART and had well-controlled HIV, we have very few IGRA results from people with antibody measurements. As a result, we are underpowered to detect a clear effect using IGRA.

From a diagnostic standpoint, both IGRA and TST are known to have reduced sensitivity in people with HIV due to HIV-associated CD4+ T cell dysfunction. While IGRA offers greater specificity in BCG-vaccinated individuals, the utility of TST using a 5 mm cutoff has been well validated in PWH and is widely accepted in clinical practice. No compelling evidence has shown that this cutoff performs substantially worse than IGRA in this population (Rangaka, Lancet ID, 2012 (doi.org/10.1016/S1473-3099(11)70210-9); Abubakar, Lancet ID, 2018, doi.org/10.1016/S1473-3099(18)30355-4 ; Luetkemeyer, Am J Respir Crit Care Med., 2007, doi.org/10.1164/rccm.200608-1088OC; Zeeb, Clin Infect Dis., 2023, doi.org/10.1093/cid/ciad330).

It is important to note that our study did not investigate diagnostic test performance but relied on the available clinical diagnostic data. Given these considerations, we believe that using combined TST and IGRA data, interpreted within the appropriate clinical context, as we did in the present study, provides a reliable categorization of MTB infection status allowing to explore the immunological consequences of MTB infection.

We accordingly made the following changes in the manuscript:

Line 204f in the methods: ”We restricted all analyses to those PWH who received an IGRA to account for the lower diagnostic precision of the TST.”

Line 271ff in the results: “We conducted a sensitivity analysis including only PWH who underwent IGRA testing (demographic details in S1 Table). Within this subgroup, we found no significant association with HIV-1 neutralization capacity (S6A Fig). Nevertheless, the findings overall suggest that asymptomatic MTB infection is associated with reduced HIV-1 neutralization capacity.”

Line 304f in the results: “We observed no effects of asymptomatic MTB on HIV-1 Ab binding reactivity when restricting to PWH who were tested with an IGRA (S7 Fig).”

**Part III – Minor Issues: Editorial and Data Presentation Modifications**

**Reviewer #1:**

Specific comments

1. Some controls are missing in my opinion (e.g. TB monoinfection or other coinfections) to know the changes in B cell response associated to. Are other than HIV specific B cell responses altered in the same way in TB-HIV co-infection?

**Reply:** We thank the reviewer for this valuable point. We fully agree that the inclusion of appropriate controls is essential to interpreting immune changes specific to Mycobacterium tuberculosis (MTB) infection in the context of HIV.

To address this, we systematically assessed the impact of several other common co-infections on B cell-related outcomes, including cytomegalovirus (CMV), hepatitis C virus (HCV), and *Toxoplasma gondii*, all of which are prevalent in people living with HIV (PWH). Importantly, while CMV was associated with enhanced HIV-specific antibody responses, this pattern was opposite to what we observed in MTB infection. In contrast, HCV and *Toxoplasma gondii* showed no association with HIV neutralization or binding, nor did they correlate with disease progression markers such as HIV viral load or CD4+ T cell counts (Fig. 1.1). These results underscore that the immunological signature we observe in the context of MTB infection is not a generic consequence of chronic co-infection but rather reflects a distinct immune modulation.

Unfortunately, MTB mono-infection could not be studied in the Swiss HIV Cohort Study (SHCS), as the cohort by definition enrolls individuals living with HIV. However, we believe the analysis across multiple comparator pathogens provides important insights into the specificity of the immune alterations associated with MTB in this population. Furthermore, both CMV and MTB are intracellular pathogens known to impact B cell function, making them particularly relevant comparators.

In another study within the SHCS, Chammartin et al. AIDS 2022 (doi.org/10.1097/QAD.0000000000003246) SARS-CoV-2 binding in PWH was assessed after mRNA vaccination in a subanalysis and found no association with MTB status, indicating, that the immune modulatory effect is specific in the HIV context.

Taken together, these findings support our interpretation that the observed changes are specific to MTB infection in the context of HIV, and not a general feature of co-infection or immune activation.

2. Reference is required in line 82

**Reply:** Thank you for noticing this oversight. We added the following references:

Davies et al. Nat Microbiol. 2024 (doi.org/10.1038/s41564-024-01678-x)

Nziza et al. Front Immunol. 2022 (doi.org/10.3389/fimmu.2022.856906)

Grace et al. Front Immunol. 2021 (doi.org/10.3389/fimmu.2021.679973)

3. Some biological measures will be described, such as immunoglobulin levels and isotype distribution. Circulating B cell levels and B cell repertoire must be described.

**Reply:** We thank the reviewer for highlighting the importance of assessing B cell biology in the context of our study. While our current work focuses on the humoral immune response as reflected in immunoglobulin levels and functional antibody activity, we agree that circulating B cell subpopulations and repertoire analysis offer crucial insights into the immunological landscape in PWH.

Although B cell phenotyping and repertoire sequencing were not within the scope of the current study, we note that detailed work on this topic has been performed previously in the Swiss HIV Cohort Study. Specifically, Liechti et al. JEM, 2019 (doi.org/10.1084/jem.20181124) used high-dimensional flow cytometry to characterize B cell perturbations in HIV-1 infection, identifying substantial shifts in naive and marginal zone B cells, including the emergence of atypical subsets with altered activation and homing profiles. Their findings demonstrated widespread B cell dysregulation in PWH, even in those on antiretroviral therapy, and form an important immunological backdrop to our current observations.

Given these foundational data, our study extends this line of investigation by evaluating systemic immunoglobulin profiles and antibody-mediated functions in the setting of co-infection. The observed alterations in IgG3 levels, in particular, add a novel layer to the evolving understanding of how chronic infections shape humoral immunity in PWH.

We fully agree that integration of B cell repertoire data with immunoglobulin and functional profiles would be highly informative, and we are planning such multi-modal analyses in future work.

We added the following text into the manuscript:

Line 448ff in the discussion:

“Although B cell phenotyping and repertoire sequencing were not within the scope of the current study, our findings align with and extend previous work on B cell perturbations in HIV-1 infection. Liechti et al. (55) described significant alterations in the composition and phenotype of circulating B cell subsets in PWH, including an expansion of atypical memory and CD21⁻ naive B cells. These shifts reflect chronic immune activation and impaired B cell homeostasis. Our study builds on this foundation by demonstrating that MTB co-infection further modulates humoral immunity, particularly through changes in immunoglobulin isotype distribution, such as elevated IgG3 levels. This underscores the unique immunological footprint of MTB in the setting of HIV, warranting future efforts to link B cell phenotypes with antibody functionality and disease outcomes.”

4. In Figure 2, it will be better to write viral load instead of RNA.

**Reply:** Thank you for the suggestion, we adapted the figure accordingly.

5. Better revision of published articles in the field.

**Reply:** Indeed, studies focusing specifically on antibody responses in individuals co-infected with HIV and Mycobacterium tuberculosis (MTB) are limited. However, recent research has begun to shed light on this area.

One notable study by Adeoye et al. iScience 2023 (doi.org/10.1016/j.isci.2023.106631) demonstrated that individuals living with HIV who also have active MTB disease exhibit broader and more potent HIV-1-specific antibody responses compared to those without MTB co-infection. This enhancement in the humoral response was associated with specific plasma mediators important for B cell and antibody development, suggesting that MTB disease can modulate HIV-1 antibody responses in a unique manner.

Additionally, a study by Nziza et al. Front Immunol. 2022 (doi.org/10.3389/fimmu.2022.856906) found that HIV infection is associated with compromised antibody responses, particularly in individuals with active TB. Despite this, differences in MTB-specific antibody profiles persist between latent and active TB disease, indicating that HIV-associated disruptions in humoral immunity do not completely abrogate the ability to distinguish TB disease states based on antibody responses.

In our current study, we have incorporated these findings into our discussion to contextualize our observations within the existing literature.

**We added the following sentences to the discussion:

Line 429ff:**
“In summary, our findings align with emerging evidence that MTB co-infection can modulate humoral immune responses in PWH (54). Adeoye et al. (29) demonstrated that active MTB disease is associated with enhanced breadth and potency of HIV-1-specific antibodies, linked to specific plasma mediators involved in B cell development. Similarly, Nziza et al. (29) reported that while HIV infection compromises antibody responses, distinctions between latent and active TB based on antibody profiles remain detectable.”

Line 468ff:
“Finally, our results are consistent with the concept of contextual pathogenicity as described by Tutumlu et al. (56), where the immunological impact of a pathogen is influenced by the host's existing immune environment and co-infections. In this context, MTB co-infection appears to uniquely shape the humoral immune landscape in individuals with HIV, underscoring the importance of considering co-infections in immunological studies.”

**Reviewer #2:**

None

**Reviewer #3:**

(No Response)
